# Contribution of primary motor cortex to compensatory balance reactions

**DOI:** 10.1186/1471-2202-13-102

**Published:** 2012-08-16

**Authors:** David A E Bolton, Laura Williams, W Richard Staines, William E McIlroy

**Affiliations:** 1 Department of Kinesiology, University of Waterloo, 200 University Avenue W, Waterloo, ON, N2L 3 G1, Canada; 2 Heart and Stroke Foundation Centre for Stroke Recovery, Ottawa, ON, Canada; 3 Sunnybrook Health Sciences Centre Research Institute, Toronto, ON, Canada

**Keywords:** Balance, Compensatory reaction, Postural perturbation, Fixed-support reaction, cTBS

## Abstract

**Background:**

Rapid compensatory arm reactions represent important response strategies following an unexpected loss of balance. While it has been assumed that early corrective actions arise largely from sub-cortical networks, recent findings have prompted speculation about the potential role of cortical involvement. To test the idea that cortical motor regions are involved in early compensatory arm reactions, we used continuous theta burst stimulation (cTBS) to temporarily suppress the hand area of primary motor cortex (M1) in participants prior to evoking upper limb balance reactions in response to whole body perturbation. We hypothesized that following cTBS to the M1 hand area evoked EMG responses in the stimulated hand would be diminished. To isolate balance reactions to the upper limb participants were seated in an elevated tilt-chair while holding a stable handle with both hands. The chair was held vertical by a magnet and was triggered to fall backward unpredictably. To regain balance, participants used the handle to restore upright stability as quickly as possible with both hands. Muscle activity was recorded from proximal and distal muscles of both upper limbs.

**Results:**

Our results revealed an impact of cTBS on the amplitude of the EMG responses in the stimulated hand muscles often manifest as inhibition in the stimulated hand. The change in EMG amplitude was specific to the target hand muscles and occasionally their homologous pairs on the non-stimulated hand with no consistent effects on the remaining more proximal arm muscles.

**Conclusions:**

Present findings offer support for cortical contributions to the control of early compensatory arm reactions following whole-body perturbation.

## Background

Compensatory reactions to sudden loss of balance involve the coordination of multiple body segments acting rapidly to restore stability. The mechanisms underlying these reactions likely reflect distributed neural networks ranging across spinal and brain stem regions up to higher cortical centres [1-3]. While postural regulation was historically regarded as sub-cortical in nature, more recent accounts include speculation about cortical contributions to the control of reactive balance [4-10]. In fact, even elements of neural function largely considered separate from posture, such as attention and working memory, reveal an interaction of postural control with higher executive networks [11-14]. Although a cortical role in reactive balance is now more widely accepted, there remains a perspective that the very early corrective reactions are driven primarily via sub-cortical mechanisms, and only the later phases of these reactions are significantly influenced by cortical drive [15].

Recent work in our lab revealed that temporary suppression of the hand region of primary motor cortex (M1) using a continuous theta burst stimulation (cTBS) pattern of transcranial magnetic stimulation attenuated hand muscle responses during a compensatory reach to grasp [16]. Focal suppression of rapid compensatory hand muscle activity indicated that M1 contributed to producing or at least scaling of the initial response. Importantly, this same pattern of suppression was noted when reaching in response to an auditory cue suggesting that both perturbation-evoked and auditory-cued arm actions were mediated through a similar cortical network. These results extend upon previous work by Gage and colleagues which revealed a preservation of spatial-temporal reach patterns during volitional and compensatory reaching [17]. Remarkably, these authors noted that despite similar muscle activity and arm kinematics, perturbation-evoked reaches were approximately twice as fast. One proposed explanation for this phenomenon is that heightened arousal drives the same cortical networks involved in producing a cortically mediated reach at a much faster rate [18,19].

In general, the recovery of balance can be achieved either by engaging muscles to re-establish a stable position over a fixed support base or to establish a new support base to catch a falling centre of mass (e.g. taking a step or grabbing a handrail). These strategies have been referred to as fixed support reactions or change of support reactions respectively [20]. In the two studies described above by Gage et al. (2007) and Bolton et al. (2011), subjects were perturbed while seated in a chair and prompted to grasp a secure handhold, therefore this represents a change of support reaction. Although the previous results offered support for the role of M1 in this corrective action it is notable that hand muscles were activated at a later stage in the reaching response. This is consistent with change of support actions which tend to recruit muscles in a proximal to distal arrangement during the reach to grasp [17,20]. While these previous studies have revealed cortical contributions to compensatory reach to grasp, it remains unclear whether the earliest compensatory responses are similarly mediated by cortical pathways. In order to explore the earliest compensatory reactions one must focus on the earliest muscle responses during execution of fixed support reactions.

The present study explored the role of M1 in the earliest postural reactions during a fixed arm support paradigm. Our specific goal was to investigate the contribution of M1 to the early compensatory arm response to rapid chair tilts while holding a fixed handle with both hands. By temporarily suppressing cortical excitability of the hand area of M1 (left hemisphere) we hypothesized that the very early hand response would be diminished in the right hand during a fixed support reaction. Moreover, given the established interaction between right and left M1 [21] we investigated the potential impact on the contralateral (left) hand. There is evidence to suggest that the predominant influence of M1 onto the contralateral M1 is inhibitory, thus inhibition of M1 on one hemisphere may result in a facilitation of homologous muscles in the contralateral M1 [21]. Some support for this idea comes from a recent study when faster reaction times for hand muscles were recorded when the contralateral M1 was temporarily suppressed [22]. Although present results revealed some variety in the precise way cTBS influenced muscle activity across subjects, there was consistently a focal impact of cTBS on the stimulated hand muscles (and occasionally homologous muscle pairs) while more proximal muscles were unaffected. Overall, these results suggest that cortical networks contribute to early compensatory arm reactions following whole-body perturbation.

## Results

The sequence of muscle activity following perturbation revealed that wrist flexor (WF) was the fastest responder for both the right (150.3 ± 5.8 ms) and left (149.6 ± 4.2 ms) limbs with the remaining muscles (first dorsal interosseous (FDI), abductor pollicis brevis (APB), and biceps brachii (BIC)) engaged approximately 20 ms later (FDI_R_: 169.4 ± 4.4 ms; APB_R_: 173.4 ± 5.2 ms; BIC_R_: 168.1 ± 5.1 ms; FDI_L_: 164.2 ± 5. ms; APB_L_: 166.8 ± 3.3 ms; BIC_L_: 170.6 ± 5.5 ms). This sequence of muscle onsets (i.e. WF engaged prior to BIC) is consistent with previously mentioned fixed-support reactions where there is a distal to proximal order of muscle activation. Moreover, the onset latency of about 150 ms for the fastest response muscle (WF) is much faster than the earliest muscle onsets involved with rapid voluntary movements using similar speeded-limb response paradigms [16,17]. This provides support for the notion that the presently observed data represent reactive balance responses.

In order to probe our specific research question it was important to establish the efficacy of cTBS in suppressing cortical excitability over the stimulated hand region. To this end, our results showed a significant drop of 23% (SE ± 9%) in MEP amplitude in FDI following cTBS in the 9 subjects where MEPs were collected (t_8_ = 2.56, p = 0.035).

**Figure 1:**
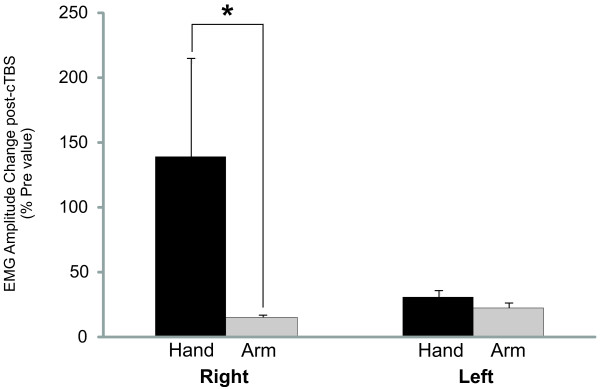
**The average change and standard error in EMG response amplitude following cTBS (i.e. difference score) is presented in bar graphs and expressed as a percentage of pre-cTBS values. **The dark bars represent hand muscles (FDI and APB combined) and the grey bars represent arm muscles (WF and BIC combined) for the right and left sides separately. * denotes significant difference at p < 0.05.

The influence of cTBS on the fixed support balance reactions was determined by comparing difference scores (post-cTBS vs pre-cTBS) which revealed a statistically significant difference in the amplitude of perturbation-evoked responses comparing between hand and arm muscles (F_1,13_ = 7.52, p = 0.018; Figure 1). Follow-up comparison revealed that Hand_R_ response amplitudes were significantly different following cTBS compared to Arm_R_, (t_13_ = 2.92, p = 0.013), but there were no significant differences when comparing left side Hand_L_ and Arm_L_ (t_13_ = 0.996, p = 0.162).

**Figure 2:**
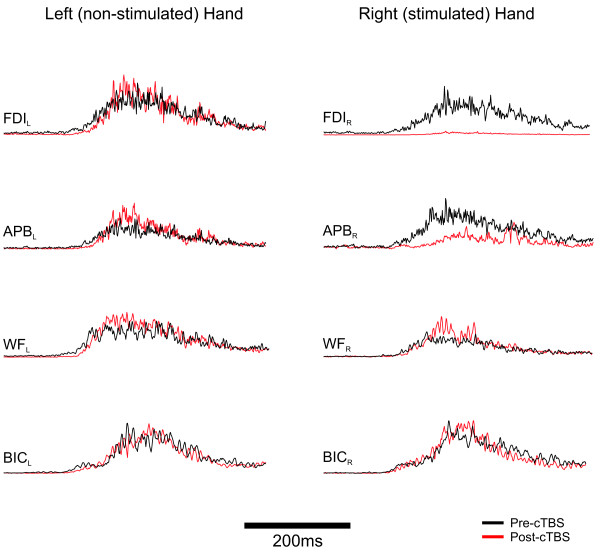
Single subject data showing both APB and FDI suppressed following cTBS.

**Figure 3:**
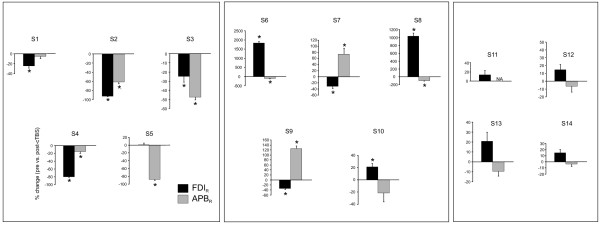
**Average and standard error EMG amplitude responses for FDI and APB in the right hand following cTBS.** Subject data grouped to demonstrate: both hand muscles suppressed (s1-5), one hand muscle suppressed while the other was facilitated (s6-10), or no significant change (s11-14). Note that all values are expressed as a percentage of pre-cTBS values and presented for each subject individually. * denotes significant difference at p < 0.05.

While a clear group difference was observed, there was inter-subject variability in the specific impact of cTBS on hand muscles which we believe may reflect, in part, the specificity of the applied cTBS. Figure 2 demonstrates an example where FDI and APB are both inhibited on the right hand, but no change in proximal muscle activation. The impact of cTBS on the stimulated hand muscles is depicted for each of the 14 subjects in Figure 3. Overall, 9 of 14 (s1-s9) subjects revealed significant suppression of the fixed support reaction in one or both of the right hand muscles (see Figure 3). In the remaining subjects there was no significant inhibition of the response amplitude in either of the muscles of the right hand.

**Figure 4:**
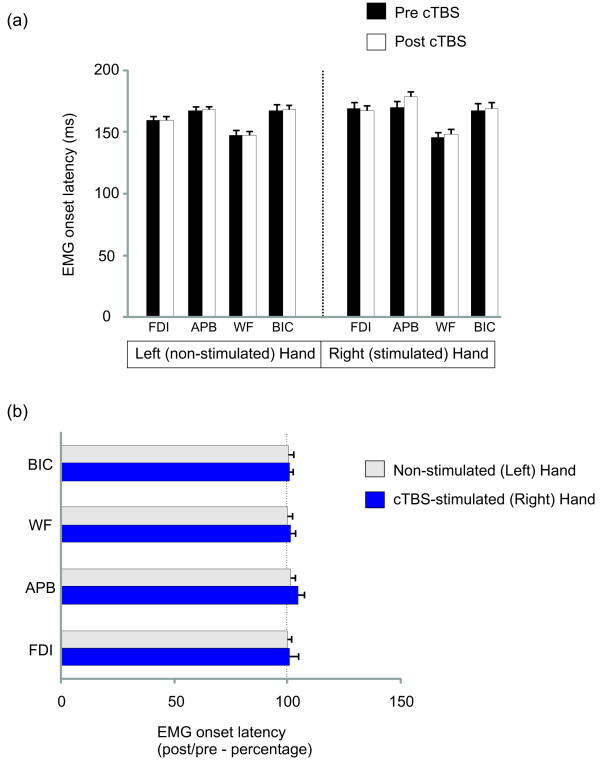
**EMG response latencies based on group average data (n = 14 subjects). **Absolute values are depicted in the top graph (**a**). The graph below expresses the response onset latencies as a percentage of pre-cTBS values to demonstrate the overall lack of stimulation effect on EMG response timing (**b**).

Comparison of EMG latencies revealed a trend for an interaction on the stimulated right arm (F_3,33_ = 2.55, p = 0.07). Follow-up comparison indicated that APB responses became marginally slower (by 8.4 ± 4.6 ms) post-cTBS in the stimulated right hand (t_11_ = 1.82, p = 0.048). There was a main effect of muscle (Right hand: F_3,33_ = 11.82, p < 0.001; Left hand: F_3,33_ =26.003, p < 0.001) with WF having the earliest response in both arms, followed by FDI and BIC at approximately the same latency, and finally APB. The onset time for each muscle is shown in Figure 4 (a). Theta burst stimulation demonstrated no significant impact on EMG onset in most of the muscles tested (the exception with a trend in APB noted above). The general preservation of response onset following stimulation for all muscles is demonstrated in Figure 4 (b) expressing post versus pre-cTBS onsets as a percentage.

## Discussion

The present study investigated the role of M1 in compensatory upper limb responses to whole body perturbation when holding a fixed support handle. To address this question cTBS was used to target cortical suppression over the hand area of the left M1 prior to the testing of perturbation-evoked compensatory fixed support reactions of the upper limb. Our results indicated that early responses of distal muscles targeted by the cTBS were indeed altered following stimulation, in the form of suppressed FDI and/or APB activity of the right hand. It is noteworthy that the influence of cTBS on these early balance reactions was often complex and evident as an interaction between FDI and APB of the same hand (e.g. a decrease in one muscle was often associated with an increase in the other). Although cross-hand interactions were not as consistently observed, there were some subjects revealing an inverse relationship between distal muscle activity in the two hands even though muscles of the left hand were not the direct target of the applied cTBS. In contrast, the proximal muscles involved in the balance reaction were not similarly influenced by the applied cTBS suggesting that the impact of cTBS was limited to the stimulated hand muscles and in some instances to their homologous muscle pairs on the non-stimulated M1. Overall, our results provide evidence for M1 in contributing to the initial fixed-support arm response following whole-body perturbation. More generally, these results provide further support for a cortical role in the control of early compensatory arm reactions.

### Cortical role in early compensatory arm reactions

Although cortical contributions to reactive balance are now more widely accepted, the stage at which cortical regions influence corrective postural activity is unclear. Findings from reduced animal preparations demonstrate the presence of direction-appropriate righting mechanisms at sub-cortical levels [23], and human studies have indicated that at least some very early postural responses are triggered prior to direct cortical involvement [24]. Therefore the fact that sub-cortical networks are capable of producing highly coordinated corrective actions, along with transit times involved in traversing cortical sites has understandably shaped thinking that early compensatory actions are not significantly determined by cortical mechanisms.

Indirect evidence suggests cortical contributions to reactive balance are not simply relegated to late-stage modifications of sub-cortically-initiated responses. As previously mentioned, Gage and colleagues (2007) observed identical motor patterns in the arm when reaching for a target handle during both compensatory and volitional reaching even though perturbation-evoked reaches were twice as fast. Remarkably, this was true despite subjects receiving the same instructions to reach as quickly as possible in both conditions. While sub-cortical mechanisms can certainly trigger rapid arm actions following a loss of balance, grasping a small target handle at a defined spatial location obviates some degree of visual guidance over the reach. Even with the prospect of descending priming over sub-cortical structures to selectively boost responsiveness [25] cortical guidance over the reach trajectory is likely needed to secure the hand onto the target handle. To explain how similar neural pathways could be engaged to produce the same reach at such an accelerated pace it has been proposed that the threat associated with whole-body perturbation heightens arousal which allows the central nervous system to overcome limits normally imposed on voluntary reaction times [18,19]. Thus the processing speed via cortical networks may be more rapid than would be normally determined from voluntary reactions. Recently, a clear role for M1 in producing or at least scaling early compensatory arm responses was confirmed more directly by Bolton et al. (2011) where cTBS was used to suppress the hand area of M1 [16]. The results of that study demonstrated suppression of the EMG responses in cTBS-targeted hand muscles of the reaching arm for both volitional and compensatory reaches offering further evidence for M1 as part of a common reaching network. Present findings extend upon the past study by revealing a similar suppression in response muscles when these muscles are engaged earlier in the response pattern (i.e. distal to proximal order with a fixed-support balance reaction).

Notably, our present paradigm focuses on the most rapid fixed support reactions that are evoked in response to postural perturbation from muscles of a limb that is already in contact with a support surface [20]. With the arms fixed onto the support handle the current paradigm is distinct from the previous compensatory reach-to-grasp, or change-in-support reactions. Fixed support reactions do not require reach to target movements that are critical to change-in-support balance reactions, such as a reach-to-grasp. With the hands already in contact with a support surface the arms not only represent a means for righting the body but also provide sensory cues for the perturbation itself. This is an important distinction from our past work given that somatosensory cues offer critical signals to trigger the initial postural reactions [26]. Studies involving rapid stretch of the thumb [27] or of ankle extensors via rapid platform translation in standing subjects [24] have demonstrated both sub-cortically and cortically mediated reflexive actions countering the imposed postural disturbance. In fact in a standing balance context Taube et al. (2006) noted cortical involvement in the leg muscle response at latencies <100 ms [24]. Although such findings relate to cortical influences over postural responses it is important to recall that in these studies there is a direct stretch of the responding muscles, thus this cortical influence could potentially reflect a trans-cortical reflex loop acting through M1. Conversely, the muscle activity measured in the present study does not specifically arise from muscles undergoing direct stretch (e.g. not simply autogenic stretch responses). Therefore sensory cues originating from a variety of body regions, including cutaneous mechanoreceptors of the hands, may provide the trigger for the multi-joint upper limb balance reactions.

### The specific role of M1 in the early compensatory arm reaction

Although our aim was to probe the role of M1 in early compensatory arm responses generally, the manner in which these responses were influenced by the applied cTBS was complex. Specifically, the interaction between FDI and APB within the same hand was unexpected. From our previous study using the change-in-support model, both hand muscles demonstrated a suppression of activity leading us to speculate that this would again be observed [16]. Presently, although some degree of suppression was common in at least one of the muscles of the stimulated hand, there were a number of subjects revealing a different relationship between FDI and APB activity. One plausible explanation for these results is the phenomenon of surround inhibition where intra-cortical inhibitory connections influence neighbouring cortical territory [28]. Surround inhibition has been demonstrated in a number of areas including M1 and has been suggested to account for discrete muscle activation thus allowing for fractionated movements of the hands [28,29]. Surround inhibition has been shown to be modulated based upon movement context and this has been specifically demonstrated between FDI and APB within the same hand [30] and has been shown to be quite variable between subjects [31]. Therefore it is possible that our results may reflect an interaction between hand muscle representations within M1, and the altered context for how the hands were used with a fixed-support versus a reach-to-grasp response may account for the variability in the present study.

An interesting finding was the relative preservation of response timing. This distinct impact of cTBS on response amplitude while leaving response onsets unaffected is consistent with our past results which focused on the change-in-support balance reactions [16]. Previously, we had noted suppressed response amplitudes in FDI and APB of the stimulated hand muscles while the remaining arm muscles were unaffected. Although the impact of cTBS on hand muscle activity is less consistent between subjects in the present study, a similar outcome was noted in that hand muscles were influenced in amplitude scaling but not onset. This may reinforce the idea that response scaling but not timing, is importantly influenced by activity within M1. Evarts noted that discharge from M1 neurons corresponded with the direction and amplitude of muscle forces required to produce movement [32]. Conversely, the selection of a motor synergy has been suggested to arise from more intermediate cortical stages of movement production such as the parietal and premotor regions [33,34]. Thus segregating the control of motor parameters in this way could result in preserving the onset of the compensatory arm pattern as a whole, while muscle amplitude is selectively diminished. Of course it was interesting to note there was a trend towards slowing of APB in the stimulated hand post-cTBS which may indicate that our present lack of influence over response timing may be a consequence of a failure to suppress M1 activity adequately below a given threshold to impact timing.

## Conclusions

Present results provide support for a role of M1 in early compensatory arm reactions to whole-body perturbation. The application of cTBS over the motor region of the distal hand muscles led to a focal attenuation on the targeted hand responses with negligible impact on the proximal arm muscles. Further, these results add to our recent data using a reach-to-grasp model and indicate that this role is apparent in both change-of-support, as well as fixed-support corrective reactions involving the upper limbs. The present fixed-support model extends previous work by revealing cortical interactions both within and between motor cortices and suggests that M1 is involved in shaping and scaling the early compensatory arm behaviour to whole-body disturbance. Future studies will address how transferable these results are to standing balance paradigms and also compensatory responses involving the lower limbs.

## Methods

**Figure 5:**
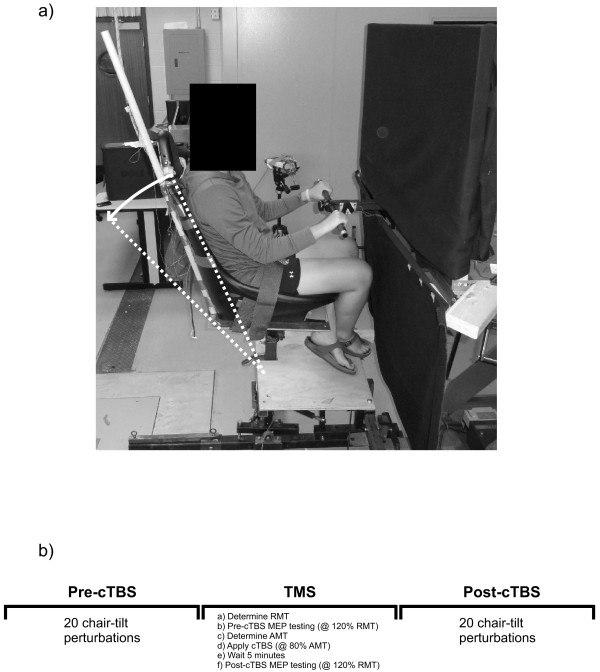
**Chair-tilt apparatus depicted in (a) (maximum chair excursion for backward tilt = 20˚).** Order of testing is presented in (**b**).

Fourteen healthy adults (9 male, 5 female; 21–38 years old) participated in this study. All participant recruitment and data collection procedures were performed in accordance with and approved by the University of Waterloo’s Office of Research Ethics. Informed written consent was obtained from all participants prior to testing (Note: consent was obtained from the subject photographed in Figure 5 to publish their picture). Participants were screened to verify that they were free of any neurological disorders prior to testing.

Participants sat in a custom-built chair that had the capability of tilting backwards in the sagittal plane to a maximum angle of 20^o^ causing a whole-body perturbation (shown in Figure 5a). The chair tilted backwards upon the release of an electromagnet (connected to a load cell), which held the chair upright. While sitting in the chair, participants were randomly exposed to temporally unpredictable perturbations. Upon receiving the perturbation participants were required to use the handle to pull themselves with both arms as quickly as possible to reposition the chair to an upright position. In between perturbations subjects were instructed to gently rest their hands on the support handle throughout testing while keeping their upper limbs as relaxed as possible. Testing order consisted of: (1) 20 pre-stimulation perturbation trials (pre cTBS), (2) cTBS applied when subjects were sitting quietly (no balance or perturbations required) followed by (3) 20 post-stimulation perturbation trials (post cTBS), for a total of 40 perturbations throughout the entire study (see Figure 5b).

Electromyography was monitored from 8 muscles of the right and left upper limbs including: first dorsal interosseous (FDI), abductor pollicis brevis (APB), common wrist flexor (WF), and biceps brachii (BIC). Before any application of Ag-AgCl electrode pairs, the skin was prepared to reduce impedance using an exfoliate (NuPrep) and then cleansing agent (50/50 H_2_0 and ethanol solution). All analog data were converted with a 16-bit, 16 channel analog to digital (A/D) converter (National Instruments, Austin, TX, USA) sampled at 1000 Hz. The chair load (i.e. tensile load via the load cell) was dual-passed filtered with a 4^th^-order Butterworth filter. The onset of the chair release was defined as the point when the chair load dropped 3 standard deviations below a resting baseline (note: this offers a conservative estimate of the perturbation onset). The EMG analyses were focused after the onset of the stimuli. EMG signals were conditioned with a bandpass (20–250 Hz) 2^nd^-order Butterworth filter, and then corrected for bias and full-wave rectified. EMG onsets were defined as the moment that signals exceeded 3 standard deviations above resting baseline for 25 ms. Average amplitude of the EMG response was calculated for 100 ms following EMG onset.

Single pulse TMS and cTBS were both targeted over the FDI representation in left M1 using a 90 mm outer diameter figure eight coil with a MagPro stimulator (MCF-B65; Medtronic, Minneapolis, MN, USA). To determine the motor hotspot for FDI in M1 of each hemisphere, the stimulator coil was positioned over left M1 and oriented 45 degrees to the mid-sagittal line to induce a current. The motor hotspot was defined as the M1 location optimal for eliciting a motor evoked potential (MEP) in the contralateral relaxed FDI muscle, Resting motor threshold (RMT) was determined as the minimum single pulse intensity required to elicit MEPs of > 50 μV (peak to peak) in 5 out of 10 consecutive trials in subjects at rest. A percentage of this value (120% RMT) was then used for collecting thirty motor-evoked potentials (MEPs) pre and 5 minutes post cTBS (in 9 of the subjects). The cTBS protocol consisted of 600 TMS pulses applied in the theta burst pattern (3 stimuli at 50 Hz repeated at 5 Hz) for 40 seconds at an intensity of 80% active motor threshold. The active motor threshold (AMT) was determined for each participant as the minimum single pulse intensity required to elicit MEPs of > 200 μV (peak to peak) in 5 out of 10 consecutive trials while subjects held approximately 10% of the maximum voluntary contraction of the FDI muscle. Next, using 80% of this value (80% AMT) we applied cTBS over the FDI target location on the left hemisphere. The procedures for cTBS are similar to those previously detailed by Huang and colleagues, [35].

Individual subject data was averaged separately pre or post-cTBS for each muscle within each arm. Response latencies were compared using two-way repeated measures ANOVA with 2 levels of testing time (pre and post) and 4 muscles (FDI, APB, WF, and BIC) for each arm separately. Significant interactions and main effects were followed-up using Tukey’s post hoc analysis. For EMG average amplitude, preliminary analysis using repeated measures ANOVA failed to reveal significant differences related to cTBS. Upon further inspection of the data it was apparent that inter-subject heterogeneity was possibly preventing stimulation effects from being detected. To contend with the variable impact of stimulation on muscle responses, initial analysis was focused on comparing the absolute difference between pre and post-cTBS values. For this comparison, all response amplitudes for each muscle were first normalized by converting them into an absolute difference score (absolute difference score = |post cTBS value – pre cTBS value|/pre cTBS value). Moreover, to account for the possibility of stimulation effects on either the targeted FDI muscle and/or the adjacent APB representation, these muscles were grouped into a common ‘Hand’ representation for the right and left hands separately. Similarly, the WF and BIC were combined into an ‘Arm’ representation for each arm separately since we hypothesized proximal muscles would remain unaffected by cTBS. These groupings allowed us to compare the impact of stimulation on the target hand area versus the non-targeted proximal arm muscles. For analysis, two-way repeated measures ANOVA was used to compare the difference scores with two levels of muscle group (Hand versus Arm) and two levels of side of body (Right versus Left side). Significant effects were followed-up using paired *t*-test comparisons. Pre and post-cTBS MEP values were compared with a paired *t*-test. Data were transformed prior to analysis if they violated assumptions of normality. Significance was set at p < 0.05 for all comparisons.

## Competing interests

The authors declare that they have no competing interests.

## Authors’ contribution

DAEB, WRS, and WEM provided concept/idea/research design. DAEB, LW, WRS, and WEM contributed to writing. DAEB and LW provided data collection. DAEB and LW provided data analysis. DAEB, WRS, and WEM provided project management. WRS, and WEM provided facilities/equipment and institutional liaisons. WRS and WEM provided consultation (including review of manuscript before submission). All authors read and approved the final manuscript.
